# Stress mapping reveals extrinsic toughening of brittle carbon fiber in polymer matrix

**DOI:** 10.1080/14686996.2020.1752114

**Published:** 2020-05-12

**Authors:** Hongxin Wang, Han Zhang, Kenta Goto, Ikumu Watanabe, Hideaki Kitazawa, Masamichi Kawai, Hiroaki Mamiya, Daisuke Fujita

**Affiliations:** a Research Center for Advanced Measurement and Characterization, National Institute for Materials Science, Tsukuba, Ibaraki, Japan; b International Center for Young Scientists, National Institute for Materials Science, Tsukuba, Ibaraki, Japan; c Research Center for Structural Materials, National Institute for Materials Science, Tsukuba, Ibaraki, Japan; d Systems and Information Engineering, University of Tsukuba, Tsukuba, Ibaraki, Japan

**Keywords:** Stress, AFM, indentation, CFRP, Atomic force microscopy, 104 Carbon and related materials

## Abstract

We conducted an in situ study on CFRP fracturing process using atomic-force-microscopy-based stress-sensitive indentation. Tensile stress distribution during fracture initiation and propagation was directly observed quantitatively. It led to a discovery that previously believed catastrophic fracture of individual carbon fiber develops in a controllable manner in the polymer matrix, exhibiting 10 times increase of fracture toughness. Plastic deformation in crack-bridging polymer matrix was accounted for the toughening mechanism. The model was applied to explain low temperature strength weakening of CFRP bulk material when matrix plasticity was intentionally ‘shut down’ by cryogenic cooling.

## Graphical abstract

**Figure:**
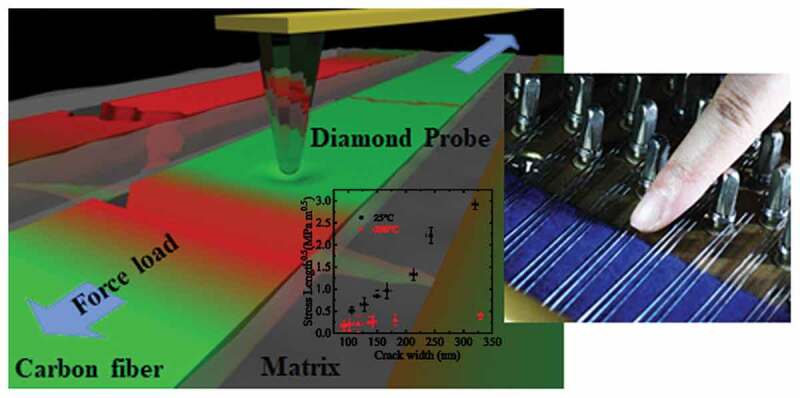

## 1. Introduction

The high stiffness and high strength per body weight offered by carbon fiber reinforced polymer (CFRP) has gained ever increasing applications for this structural composite in areas of astronautics, aviation, automobiles, ships, infrastructure constructions, sports goods, etc. More than 50wt.% of large passenger aircraft are now made by CFRP [1]. While a simple law of mixture can explain the high stiffness of CFRP coming from the high stiffness of carbon fiber with analogy to springs in parallel, the mechanism for the composite high strength is not trivial [2]. Though individual carbon fiber has a high strength, Griffith discovered 100 years ago that small fiber of brittle material is stronger than its bulk counterpart only because of the lower chance to develop a critical fracture-initiating defect [3]. It would then be deduced that such statistical advantage of small fibers should vanish due to averaging effect when large amount of them is used to form a bulk material. Fracture mechanics following Griffith’s study also established that the strength of brittle materials, including CFRP, is determined by the property of fracture toughness, which is the material ability to resist fracture propagation [4]. CFRP exhibits fracture toughness almost twice that of both constituents: the strong but brittle carbon fiber (CF) and the ductile but weak polymer (P) matrix, thus breaking the law of mixture [5–7].

Further increasing the toughness, thus the strength, of CFRP, especially under various environmental conditions, remained as one of the biggest effort-concentrating fields in material research. Despite research efforts for over 40 years, the microscopic mechanism of CFRP toughening remains unclear. At unidirectional ply level, models widely used by scholars all assumed CFRP failure process starting with catastrophic fracture of individual CFs at positions and stress levels of statistical nature [8–10]. The toughening of composite was believed to be a result of reduced stress concentration on intact CFs adjacent to a fractured CF through a complex interaction between both P matrix and CF/P interface [11]. The fact that mechanical properties of CF/P interface being not directly measurable leads to inconsistent CFRP strength predictions throughout literature. For example, when CFRP-constructed large passenger aircraft are at their cruising altitude, environmental temperature drops to −60°C. The phenomenon of low temperature weakening of CFRP material demands a fundamental mechanism understanding to guide improvement. Existing model suggests that CF/P interface weakening was the cause, while other studies showed clear strength increase for both P matrix and CF/P interface as temperature decreases [12–14]. Therefore, it is of priority to scrutinize the microscopic process of CFRP fracturing/toughening using in situ direct stress characterization with spatial resolution adequate to resolve a single nanoscale fracture-initiating defect. Current methodologies, such as Raman spectroscopy, X-ray diffractometry, and electron/optical microscopy, lack either nanoscale resolution or stress sensitivity for CFRP bulk material [15–21]. The characterization challenge is exacerbated by the distinctively different physical properties of CF and P, which excludes methods suitable for only one constituent. For example, the fact of CF being conductive, semicrystalline, and opaque while P being insulating, amorphous, and transparent, hinders both electron and photon beam-based stress-sensing techniques [22].

In this work, we used atomic force microscopy (AFM) indentation to point-by-point characterize the CFRP local tensile stress, which is defined as the stress value averaged over the specimen area covered by an indentation point. The new technique revealed that nanoscale fracture in brittle CF develops in a stable manner with increasing load, which was considered only possible with ductile materials capable of plastic deformation. Energy dissipation through yielding of the adjacent P matrix is accounted for the observation. It acts through an extrinsic toughening mechanism similar to biological systems [23,24]. To prove this mechanism, we intentionally ‘shut down’ such extrinsic-toughening by cooling the specimen to cryogenic temperatures. CF fracture returned to brittle catastrophic manner. Bulk strength of CFRP also significantly decreased. Our observation countered the previously believed significance of stress concentration reduction in CFRP toughening. It provides hope for further improving CFRP toughness/strength by controlling fracture propagation at subfiber level through matrix plasticity engineering.

## 2. Materials

Polyacrylonitrile (PAN)-based high strength standard modulus carbon fiber (T700s) provided by Toray company was used for this study. The CFRP material used in this study is a unidirectional composite T700S/2592. It consists of the high strength carbon fiber T700S and an epoxy resin 2592 with the cure temperature of 130°C. The unidirectional carbon/epoxy laminates were fabricated from the prepreg tape of P3252-20 (Toray, Japan). They were laid up by hand and cured in an autoclave. The glass transition temperature of the epoxy resin in the laminates was about 100°C. CFRP samples with size of 30 × 3 × 1.5 mm were cut from bulk using diamond coated disk saw. Sample surface was then sanded which is followed by multistaged polishing with final finishing using SiO_2_ abrasive with 50 nm particle size.

## 3. Methods

**Figure 1.**
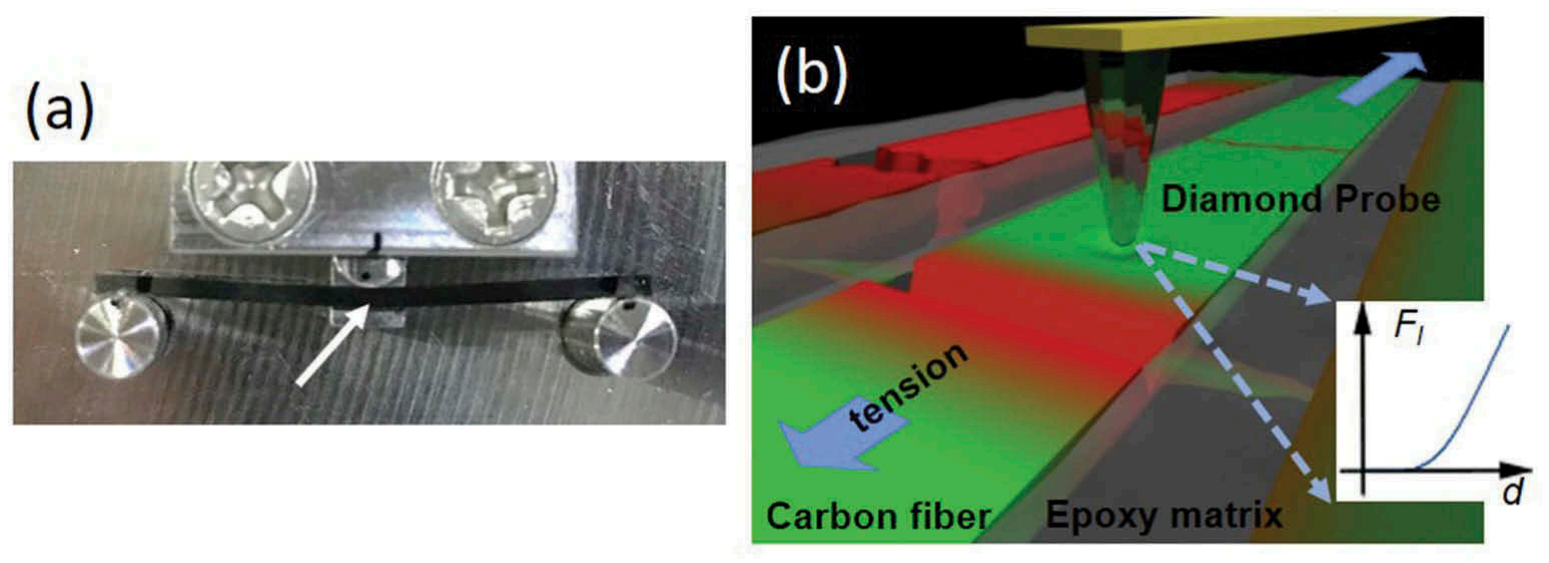
(a) Photograph showing the three-point bending stage. Area marked by the white arrow is subjected to AFM pinpoint indentation; (b) Illustration showing the mechanism of pinpoint indentation. Inset showing recorded *F_l_-d* curve where x-intercept is used to construct height map

The CFRP sample was mounted on a three-point bending stage, as shown in Figure 1a. The actuator in the middle pushes the bar-shaped sample to generate tensile stress in the area marked by a white arrow. This area is characterized by AFM. (Pinpoint mode by NX10, Park System, Korea) The principle of AFM pinpoint indentation is illustrated in Figure 1b. A sharp diamond probe with a tip radius *R* of 10 nm was used to perform nanoscale indentation point-by-point on the CFRP surface [25]. With indentation force *F_l_* (1000 nN in this case), local specimen deformation *d* could be recorded for each scanned position. One example of such a recorded *F_l_-d* curve is shown as the figure inset. Hertz model of contact mechanics was used to convert the local deformation into reduced indentation modulus *E* [26,27]:

(1) $$E=\frac{3F_{l}}{4\sqrt{R\times d^{3}}}$$

Topographic height map (z map) is simultaneously created for each indented spot using the stage height value upon probe-specimen contact (x-intercept in Figure 1b inset).

Stress calibration on CF was performed by placing the same CFRP sample-bending stage under the objective of a confocal Raman microscope (RAMANPlus, Nanophoton, Japan). Same area as that characterized by AFM was analyzed by Raman spectroscopy. Stress value in the CF was obtained by Raman spectroscopy. In the same time, actuator force and displacement were also recorded to calculate an average stress at position of Raman characterization.

## 4. Results and discussion

**Figure 2.**
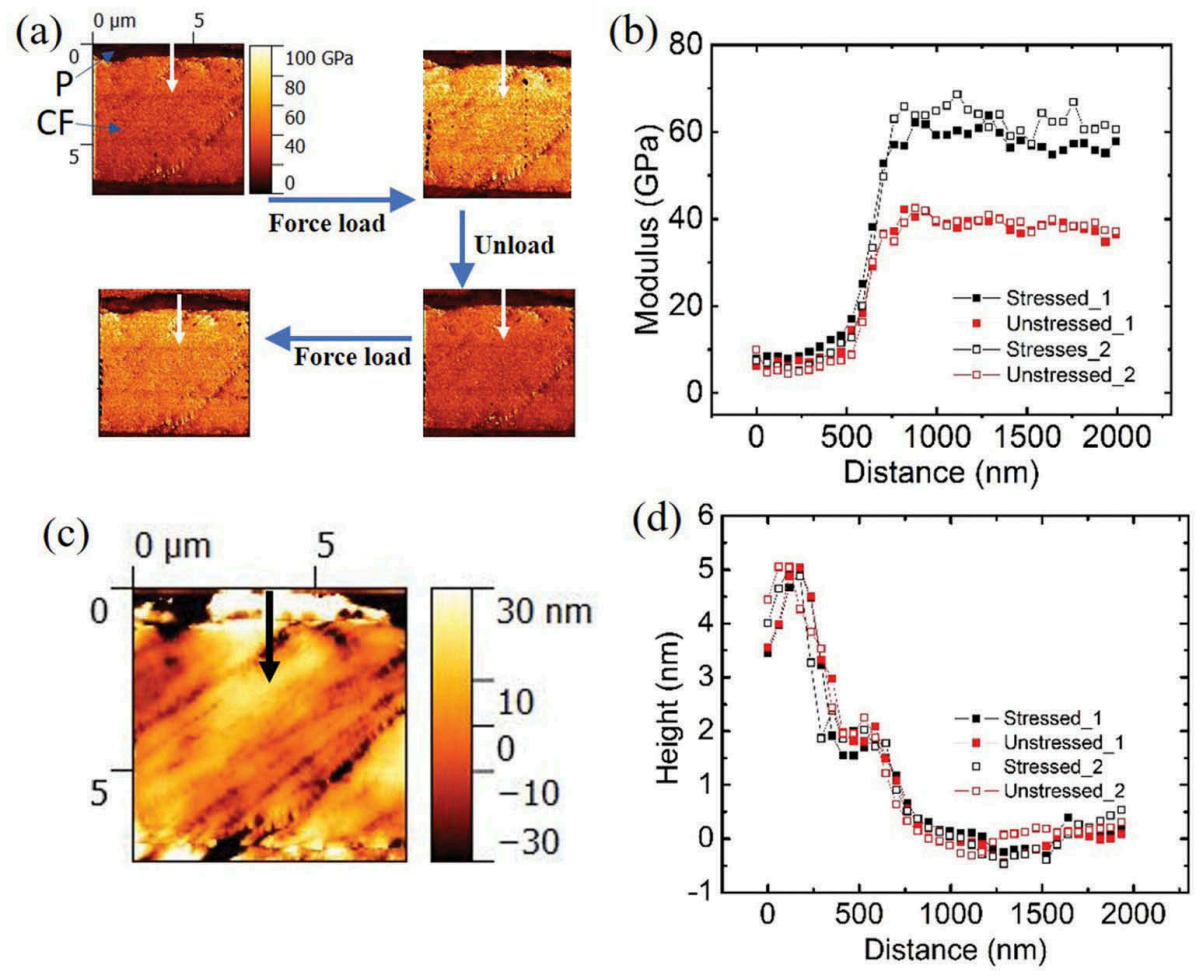
(a) Indentation reduced modulus maps created for the same CFRP sample region during load/unload cycles with tensile stress of 1.2GPa; (b) modulus line profiles created along the white arrows marked in (a); (c) a typical height map of the same region in (a) with a black arrow marked in the same position as the white arrows in (a); (d) height line profiles created along the black arrow in (c)

### 4.1 Stress mapping of CF

It was reported in several AFM indentation studies that the measured value of E is affected by the presence of in-plane tensile stress [28–30]. Therefore, it is possible to extract the stress value from the change in apparent E values when specimen is measured before and after tensile load. Figure 2a displays a series of apparent indentation *E* maps created about the same specimen area with and without the tensile stress of 1.2 GPa, which is generated by the three-point bending method. The up-left panel shows an unstressed CFRP with CF modulus averaged around 44 GPa and P modulus averaged around 8 GPa. The indentation modulus value obtained on the CF longitudinal intersection in a previous report is ~33 GPa for experiment and ~25 GPa for FEM simulation. The CF used in that study (T300) and ours (T700) are both PAN-derived type with similar tensile moduli (~230 GPa vs. ~250GPa) [31]. The 33% higher indentation modulus can be explained by the much smaller indentation deformation used in our case (<10 nm) compared with theirs (~150 nm). It is known that CF indentation modulus decreases dramatically with increasing deformation due to buckling of graphitic layers [32]. Upon bending, the average *E* as measured for CF became 77 GPa. The *E* for CF could return to low value after removing the external load and this cycle could repeat with good consistency, as shown in Figure 2a. Figure 2b is composed of line scan profiles created along the white arrows marked in the *E* maps of Figure 2a. The line scans start from a smaller region of P towards inside of CF. It is clearly shown that the *E* values for both CF and P increase with increasing stress. It is known that specimen topography, such as tilting angle, influences indentation modulus [33]. The simultaneously recorded four height maps were carefully compared and confirmed that they are identical without external load dependence. One such height map is displayed in Figure 2c. Line scan profiles on the same region are displayed in Figure 2d. Black arrow indicates the line scan region. The possibility of topographic dependence on tensile load is thereby excluded.

**Figure 3.**
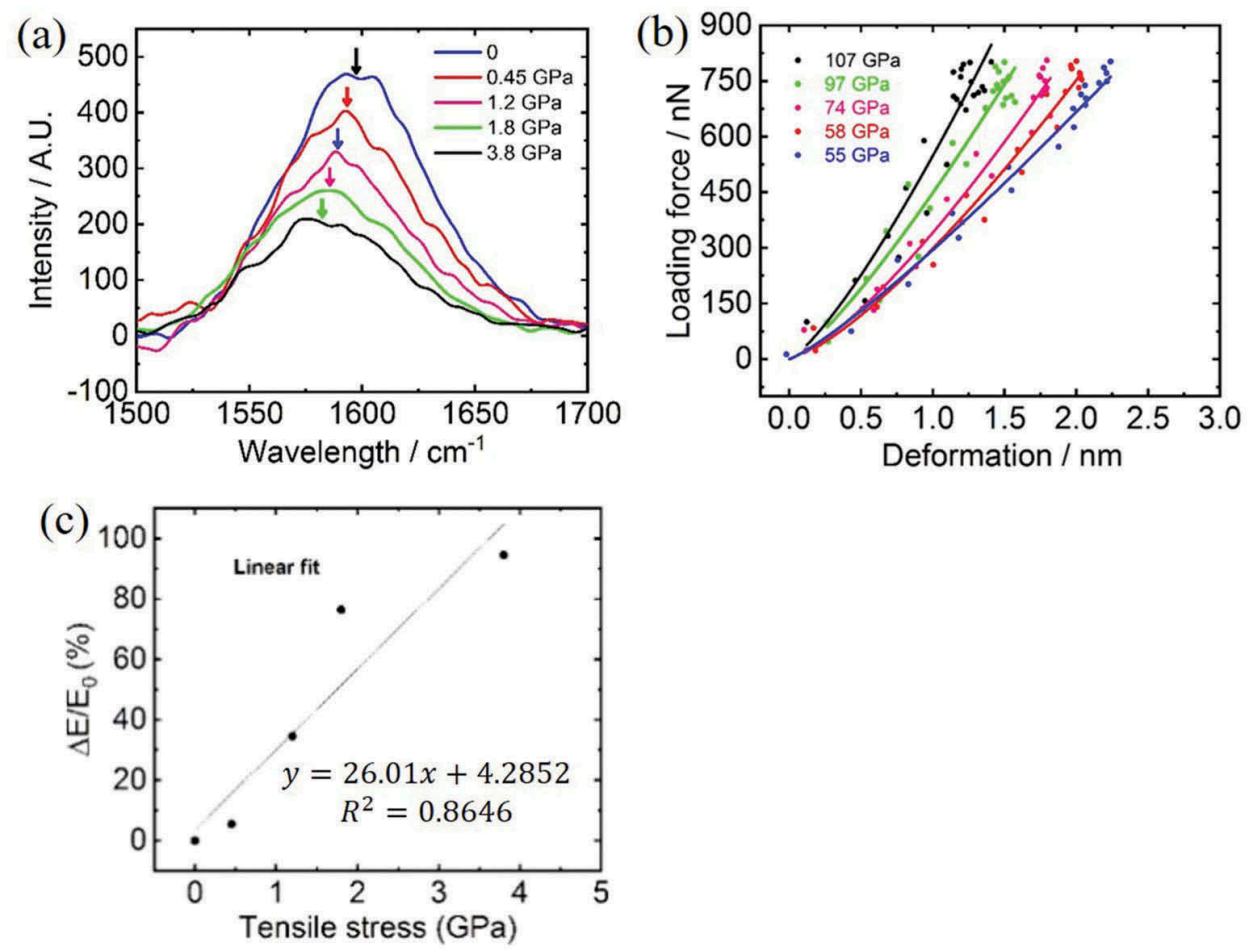
(a) Raman spectra recorded on the same carbon fiber under five different tensile stresses; (b) force-deformation curves recorded by using AFM on the same carbon fiber under the same tensile stress in (a); and (c) correlation between the relative change of indentation moduli and the tensile stresses as calibrated by Raman spectroscopy

For stress calibration, we collected Raman spectra on CF for each bending conditions and the averaged spectra are displayed in Figure 3a. The red-shift of 1590 cm^−1^ peak in response to tensile stress has been extensively studied in literature for PAN-based CF [34]. We adopted the shift rate of 1.8 cm^−1^/GPa to calibrate stress used in our experiments. *E* maps were created for the same CF region with conditions of no stress and four increasing stresses. Typical force-deformation (*F_l_-d*) curves representing five modulus maps are displayed in Figure 3b. The indentation modulus *E* obtained from curve fitting with equation (1) increases with increasing tensile stress. The stress-response of *E* could be explained by a model based on stressed surface [28–30]:

(2) $$\frac{\Delta E}{E_{0}}=\frac{E-E_{0}}{E_{0}}=\frac{1}{\frac{F_{l}}{\beta \Delta \sigma }-1}$$

Where *E_0_* denotes unstressed indentation modulus; *σ* denotes stress in the surface; *β* is a coefficient related to indentation deformation. When β⋅σ is much smaller than *F_l_*, the relative modulus change Δ*E*/*E_0_* forms linear relationship with membrane stress *σ*. We plotted Δ*E*/*E_0_* against the Raman calibrated applied stress values in Figure 3c. Δ*E*/*E_0_* was found roughly with a linear relation with tensile stress forming a gradient of 26GPa^−1^. This linear response was then used as basis for following quantitative analysis of CFRP fracture propagation.

**Figure 4.**
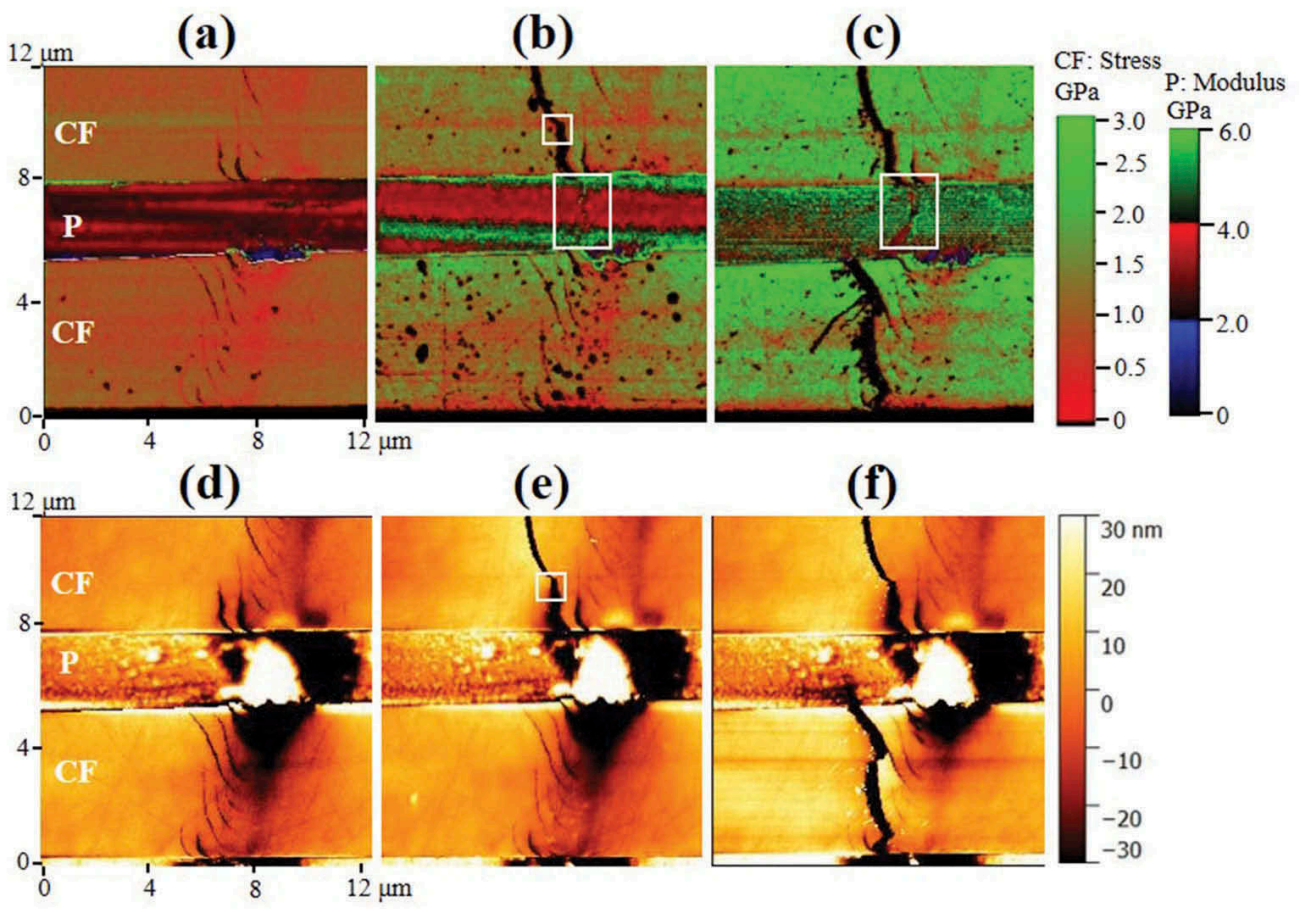
(a–c) Local tensile stress maps created for a CFRP specimen under tensile stresses of 0, 2.4, and 4.8 GPa, respectively; (d–f) height maps recorded simultaneously with modulus/stress maps shown in (a–c)

### 4.2 In situ *stress characterization of CF fracturing*

Figure 4a–c are three stress distribution maps converted from the corresponding CF modulus maps using the conversion relation of Figure 3c. In P region, indentation modulus is used as it is without conversion. CF region and P region are displayed with separate scales with left stress scale for CF and right modulus scale for P. Figure 4d–f are the corresponding height maps for 4a–4 c respectively. Figure 4a displays the original state for the double CF region before bending. Several shallow surface defects with ~10 nm in depth are visible on both CFs which are bridged by a hill-and-valley region in the connecting polymer matrix. Those are likely caused by the sanding/polishing treatment during sample preparation. Figure 4b and e present the stage where the initial defect in the upper CF developed into a crack. It is interesting to notice that a line of higher stress formed in the P matrix below the upper CF crack, as marked by the lower white rectangle in Figure 4b. Figure 4c and f present the state where the stress transferred from the upper CF crack induced a new crack in the lower CF. The higher stress line marked by the bigger rectangle in Figure 4b has developed into a region where stress is lower than surroundings, as marked by the bigger rectangle in Figure 4c. It suggests matrix yielding where local tensile stress stopped increasing with increasing strain and elastic energy is consumed by plastic deformation [35].

**Figure 5.**
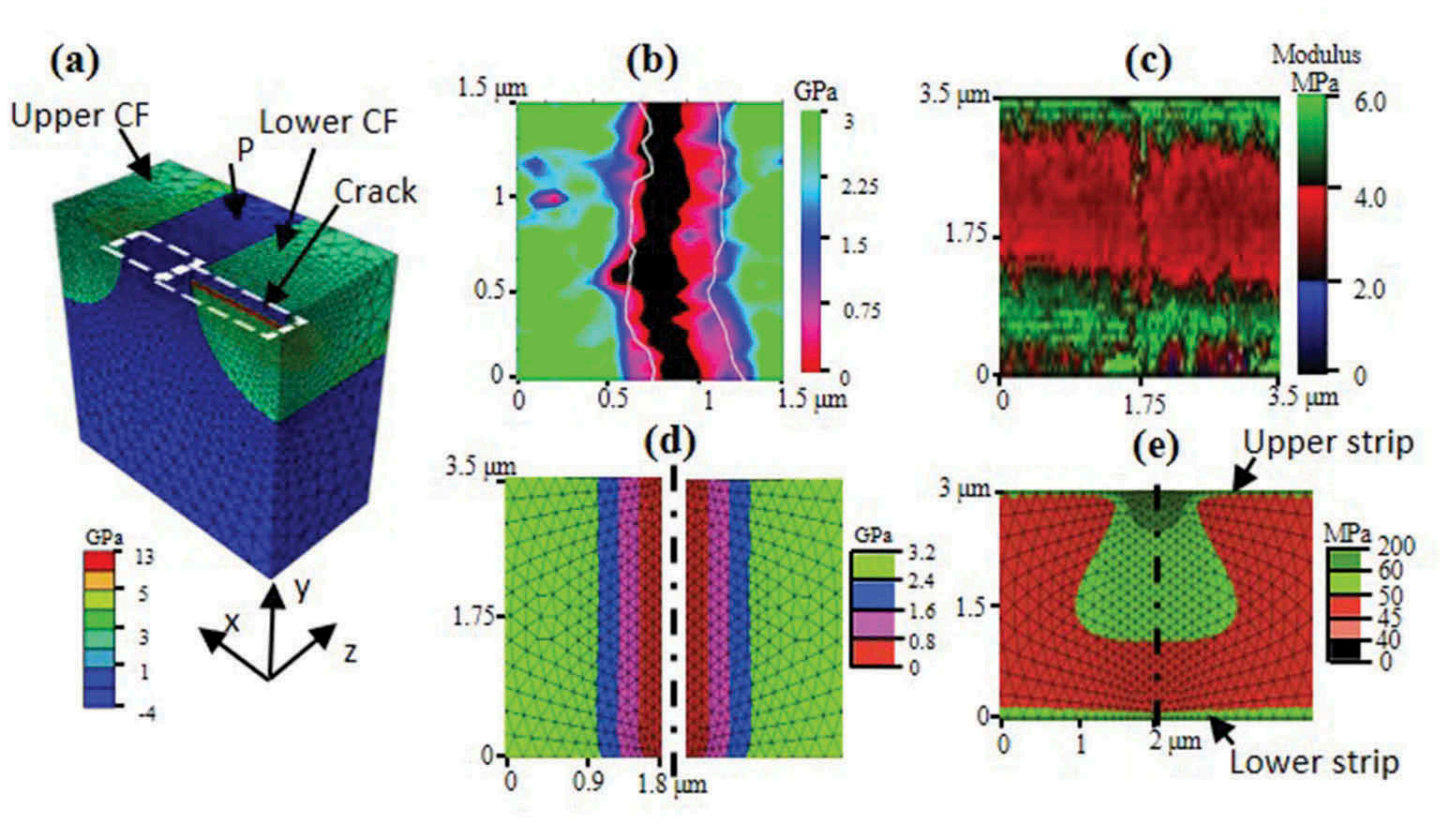
(a) Finite element model created to simulate a polymer matrix containing two carbon fibers with one intact and one cracked only on its surface; (b) magnified and rescaled stress map for the region marked by the upper rectangle in Figure 4b. White solid lines marked crack edges as measured in height map of Figure 4e; (c) magnified modulus map for the region marked by the lower rectangle in Figure 4b; (d) a mirrored maximum principal stress map simulated from the region marked by the right rectangle in (a); and (e) a mirrored maximum principal stress map simulated from the region marked by the left rectangle in (a)

### 4.3 FEM simulation on CFRP stress distribution

Finite element simulation was used to verify this hypothesis with a model shown in Figure 5a. The modeled contains two CFs and the interconnecting P matrix. The upper CF is fixed at all surfaces while the lower CF is fixed at all surfaces except its top surface, which is displaced towards tensile load direction (z direction) by 125 nm. (see supplementary materials for more details) Maximum principle stress distribution on the model top surface is displayed for both CF and P. Two areas marked by white rectangles are highlighted as Figure 5d and e to compare with experimental result of Figure 5b and c. Figure 5b is magnified rescaled *E* map of the region marked by the upper rectangle in Figure 4b. Contour lines showing the broken edges of the CF parts are also displayed in the same graph. It is apparent, especially on the left side of the broken edge, that stress magnitude started to drop before the drop of height due to scanning probe tip shape. The gradient zone before the edge could be as long as 500 nm, which is more than 20 times the diameter of the probe tip. It is known in a fiber reinforced composite that stress on a completely broken fiber is rebuilt to form a stress gradient over a distance called ineffective length [36]. The minimum ineffective length assuming a perfectly bonded interface is larger than 8 times the diameter of the fiber, which would be longer than 60 μm in our case [37]. As can be seen in Figure 4b, the stress on the broken fiber is almost the same as the lower intact fiber, except for the 500 nm gradient zone shown in Figure 5b. A partially broken fiber with a stabilized crack growth is therefore suggested. Figure 5c is a magnified view of *E* map on P area marked by the lower rectangle in Figure 4b. Figure 5d is a stress map on the surface outside of the crack and mirrored for better visualization. It shows that the created surface crack does generate a localized stress gradient within 800 nm away from the crack edge. There is also a good match between the simulated stress map of Figure 5e and the measured stress map of Figure 5c about the P area adjacent to the crack. Besides the central high stress area just below the crack, there are also two horizontal strips of high stress region along the z direction at the CF-P interfaces, with the lower strip wider than the upper strip. The double strip feature is a good match with those observed in Figure 5c.

### 4.4 CF toughening mechanism

Estimation of fracture energy could be carried out by considering modified Griffith’s criteria [3]:

(3) $$\sigma_{f}\sqrt{a}=\sqrt{\frac{EG}{\pi }}$$

Where *σ_f_* is fracture stress; *a* is fracture depth; *E* is Young’s modulus of carbon fiber and *G* is total energy increased during fracture growth. *σ_f_* equals applied stress and *a* can be measured from height maps in Figure 4. We then could obtain the total energy *G* of the cases in Figure 4a and c respectively. With a crack depth of 13 nm, *G* becomes 0.13 J/m^2^ for Figure 4a, where no plastic deformation in P is observed. Assuming a pure brittle fracture, the surface energy for CF is then half of *G*, as 0.065 J/m^2^. It agrees quite well with ~0.04 J/m^2^ for similar CF in literature [38]. The case in Figure 4c gives *G* value of 19 J/m^2^, which cannot be explained by surface energy alone in the case of brittle fracture. Therefore, energy dissipation through plastic deformation must be considered. However, slit tests were used to study individual CF fracturing process in literature and they all reached the same conclusion of typical brittle fracture without plastic deformation. Only epoxy matrix could be the source for the elastic energy dissipation induced by plastic deformation. G obtained from Figure 4c is however 1 order of magnitude smaller than typical fracture energy reported for epoxy blends [39]. It indicates that the toughening mechanism observed here might not be conventional intrinsic toughening where plastic deformation zone is ahead of the crack opening. It suggests an extrinsic type where plastic deformation functions behind the crack opening as crack bridging [40]. A recent study indicated that even brittle epoxy matrix could undergo equivalent plastic strain as large as 50% without losing stiffness under appropriate load condition [41].

**Figure 6.**
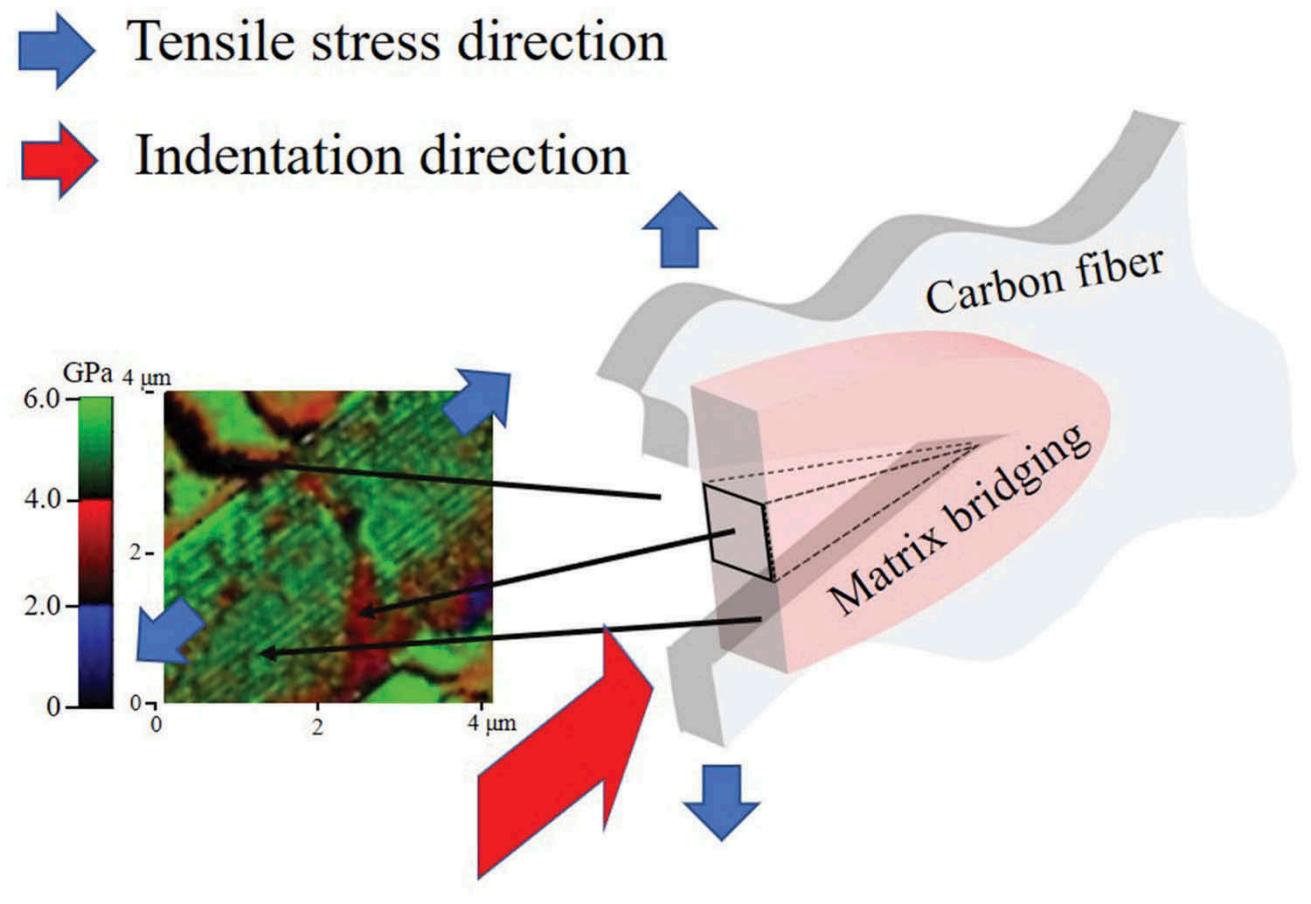
Illustration of the extrinsic toughening mechanism accounted for the stable fracture growth inside of a carbon fiber with upper, middle, and lower black arrows pointing at CF crack opening, P plastic deformation zone, and P elastic deformation zone respectively; Stress map on the left with one-to-one correspondence with zones labeled in the model

An explanation of the observed toughness is thus proposed. The increased stress enlarged the opening of the fracture as shown in Figure 6. The release of CF elastic energy is in competition with thermal energy conversion of the bridging P plastic deformation, as evidenced by the observed P yielding zone in Figure 6, which is a magnified view of Figure 4c. The fracture is stabilized and its propagation is stopped when these two energies reached balance.

**Figure 7.**
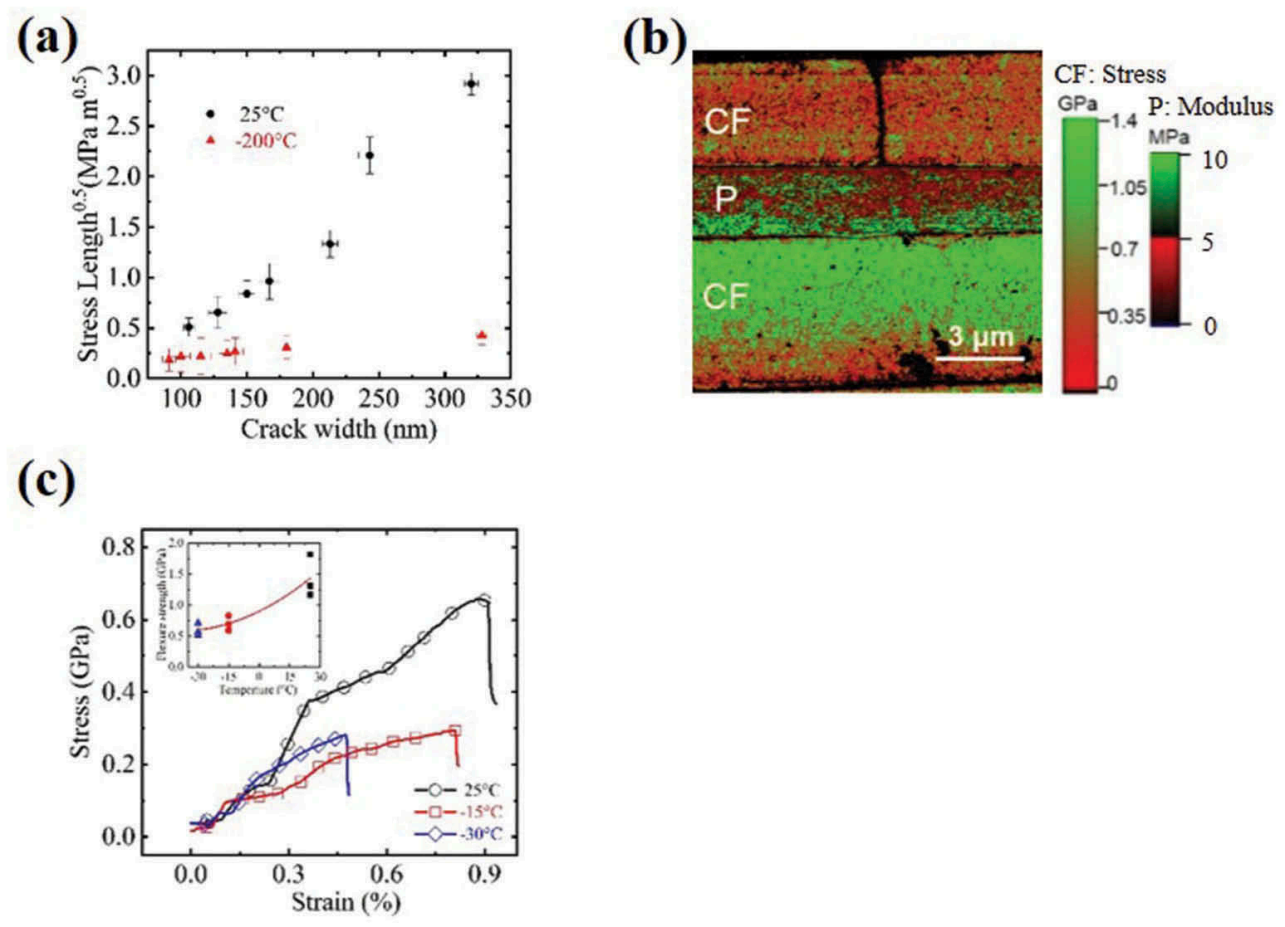
(a) Plot of fracture toughness against crack width measured at seven random fracture locations on CFRP specimens stressed at room temperature and −200^°^C respectively. (b) A typical stress map of a CFRP specimen stressed at three-point bending stress of 1.2 GPa and at −200°C. The left scale bar for CF indicates tensile stress and the right scale bar for P indicates local modulus; (c) stress-strain curves obtained by three-point bending tests for CFRP specimen at 25^°^C, −15^°^C and −30^°^C respectively; inset shows plot of ultimate strength for three specimens at each temperature

Next, crack growth resistance curve (R-curve) is used to differentiate intrinsic and extrinsic toughening mechanism by observing the trend of fracture toughness change against balanced crack extension size. Mode I fracture toughness *K_IC_* is defined as critical stress intensity factor, $\sigma_{B}\sqrt{a}$, where $\sigma_{B}$ is the applied tensile stress on CF in balance with the crack depth and *a* is the depth of crack. Since the crack depth measurement by AFM is heavily influenced by the tip size and shape, *a* is therefore taken as crack width which can be accurately measured from AFM height maps. If a fixed ratio between a crack depth and width is assumed, such approximation will not change the trend in an R-curve even though the absolute *K_IC_* values could be underestimated. Altogether seven randomly located fracture sites were analyzed for a CFRP specimen and their calculated *K_IC_* were plotted against crack width in Figure 7a. A clear rising R-curve is evident to support an extrinsic toughening mechanism. The situation is similar to biological material toughening in natural nacre or bones, which are also composites of brittle calcium carbonate and ductile proteins [40]. To further verify the proposed mechanism, the effect of the P matrix plasticity was intentionally ‘shut down’ by cooling the CFRP specimen to low temperature. After applying bending load to specimens at −200°C, they were naturally warmed to room temperature before characterization by AFM indentation. A typical stress map for a CFRP deformed at −200°C is shown in Figure 7b. The upper CF with a crack is found with a much lower stress value compared with the intact adjacent CFs in the same image, showing that the crack is completely fractured through its thickness so that no stress could be carried on the CF near the fracture site. The existence of only one Poisson strip near the intact CF side in the P matrix also supports the complete loss of load carry ability of the upper fractured CF. This contrasts with the two strips observed in Figure 4b. Unlike the case where CFRP was deformed at room temperature, the crack width of the −200°C deformed specimen was much narrower. Plot with *K_IC_*, as previously defined, was made by seven random fracture locations and the results are added to Figure 7a for comparison. It clearly showed a flat R-curve with low values of *K_IC_*, which is typical for brittle CF fracture. It must be noted that the crack width measured at complete fiber fracture is larger than the critical crack width from which CF failure developed. However, it gives an upper boundary for the true critical crack size. Nine CFRP specimens were subjected to bending force till complete failure with 3 specimens per testing temperature at −15^°^C, −30^°^C, and −200^°^C, respectively. Same strain rate was used so that the composite strength will only be affected by testing temperature. Typical stress-strain curves were compared in Figure 7c and the reduction of both ultimate strength and strain with decreasing temperature was evident, agreeing with previous reports by others [12,42]. Our characterization and analysis are all based on surface phenomenon. Both AFM and Raman spectroscopy detects only stress on the surface. We also based our discussion on surface crack and polymer yielding zone on the surface. These surface observables are adjacent to each other and showed clear correlations. So, we proposed the mechanism revealing the interplay by these surface observables. However, it should also apply in bulk form, because the mechanism itself does not involve surface-only phenomenon. Moreover, it is likely that, even in the CFRP body, CF crack and P yielding occur first on their surface/interface.

**Figure 8.**
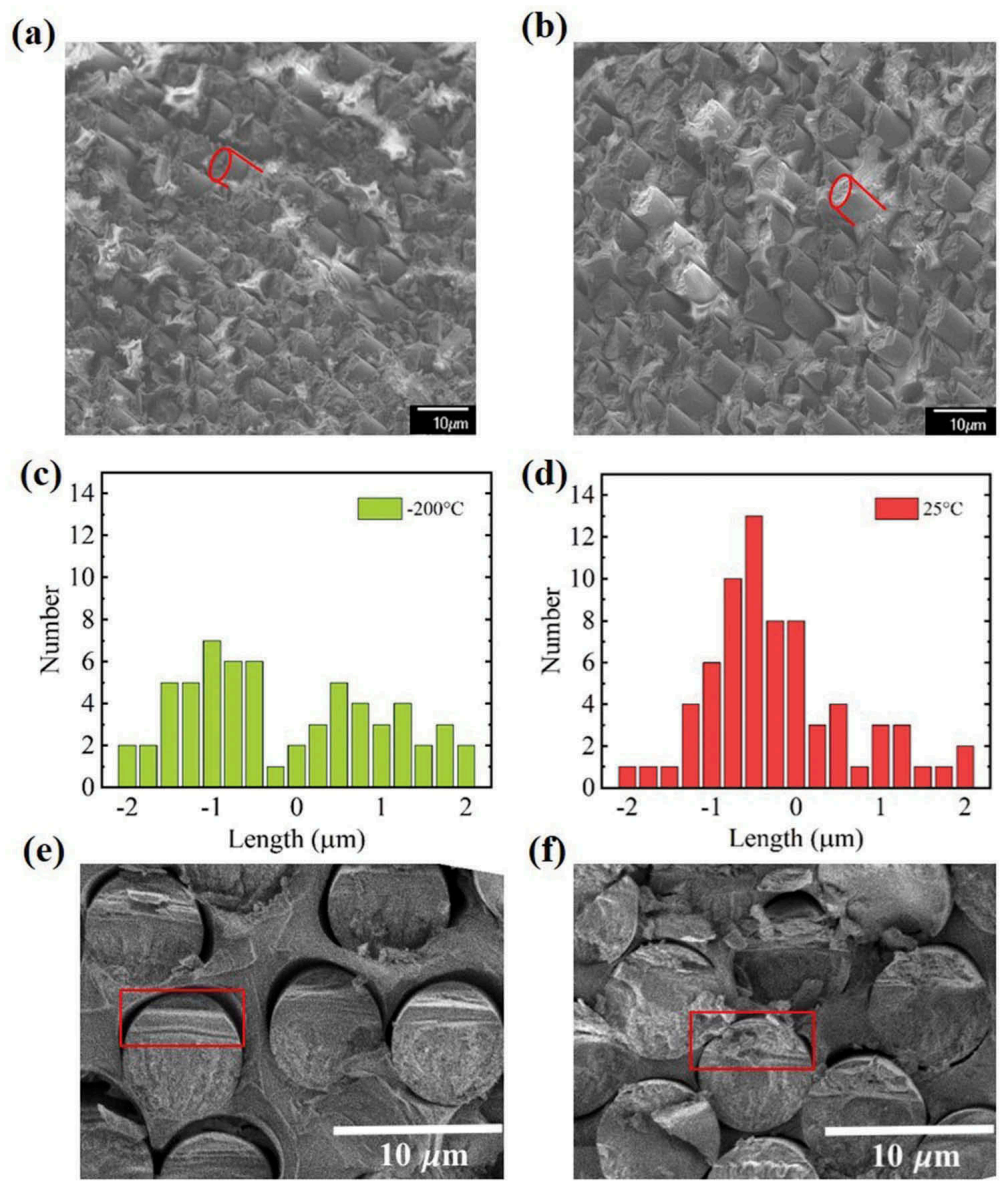
(a, b) SEM images taken for specimens fractured at −200°C and 25°C, respectively; Two broken CFs were outlined in red color to assist image comprehension; (c, d).Histograms of protrusion length of broken CFs in SEM images of (a) and (b); (e, f) SEM head-on shots of CF surface fractured at −200°C and 25°C, respectively

T9To test our proposed model on fracture propagation in a CFRP bulk, we compared morphology of fractured surface of specimens fractured at −200°C and room temperature. It is also intended to examine the validity of the previously believed mechanism where reduction of stress concentration is the cause for CFRP toughening. During room temperature fracturing, the surface crack observed in Figure 4b clearly propagate into the adjacent intact CF through stress concentration in the P matrix. On the contrary, for −200^°^C fracturing, a through crack observed in Figure 7b did not propagate or cause high degree of stress concentration. These observations intuitively countered the previously believed model. Moreover, we also conducted scanning electron microscopy (SEM) characterization over the fractured surface for both the specimens failed at −200^°^C and those at room temperature, as shown in Figure 8a and b. The direct effect of stress concentration is to allow fracture propagation to stay in the same direction. It implies that at the fracture surface, one should observe broken CFs out of the P matrix with similar protrusion length. Relatively flat area of about 0.01 mm^2^ which contain about 80 broken CFs was imaged for both samples. The number of CFs is plotted against the difference between individual protrusion length and the average value for the entire area, as shown in the histograms of Figure 8c and d. It is quite clear that room temperature fracture is with a more uniform ‘cut’ at the fracture surface, where CFs broke at similar positions. On the contrary, −200°C sample showed a rather random distribution of protrusion length, indicating breakage of CFs occurred rather independently based on their own statistical distribution of defects with critical size. Our observation suggested that stress concentration is less in the case of low temperature fracture, opposite to what was believed in the past. This is because in the low temperature case, defected CF did not develop high strain yet before complete fracture. Therefore, the amount of elastic energy released upon such catastrophic fracture is less than the case for stable fracture where the larger crack width generated much higher elastic deformation in the adjacent P matrix and consequently adjacent intact CFs. However, the significantly reduced toughness in the individual CF fracture caused premature failure of the whole CFRP at the lower temperatures, even though stress concentration is also reduced. SEM head-on shots of the fractured CFRP surface were presented in Figure 8e and f. In the case of −200^°^C fracture, the CF surface can be clearly divided into two zones. According to fractography of brittle materials, the upper zone, as marked by the red rectangle in Figure 8e, is with smooth surface and termed as ‘mirror zone’. The lower part of the fracture surface with higher degree of roughness is the zone of ‘mist’ and ‘hackle’ [43]. The transition from the smooth zone to the rough zone indicates that crack tip propagation velocity dropped due to a reduced driving energy. The roughness is due to formation of many small cracks along the direction of the original major crack front [44]. The appearance of the −200^°^C fractured CF surface is quite similar to focused ion beam (FIB)-notched fracture surface of the same type of CF (T700), though the latter fracturing was performed at room temperature [7]. It is quite interesting to notice in Figure 8f that the room temperature fractured CF surface, inside of CFRP, can also be divided into two zones with the upper zone occupying about 1/3 of the total cross-section. However, though the lower zone is with similar degree of roughness as that in Figure 8e, the upper zone is much rougher, as marked by the red rectangle in Figure 8f. The CF fractured at room temperature inside of CFRP is observed lack of a mirror zone with high crack propagating velocity. It is therefore deduced that when crack started inside of a CF in CFRP at room temperature, additional energy consumption occurred, which resulted in less crack-driving energy and consequently lower propagation speed. This observation agrees well with our proposed model where CF crack elastic energy is consumed by plastic deformation of the P matrix during fracture propagation.

## 5. Conclusions

Calibrated using Raman spectroscopy, AFM pinpoint indentation provides a quantitative in situ approach to characterize CFRP stress distribution. The tensile stress distribution was characterized during propagation of a CF fracture to neighboring CFs. An extrinsic toughening mechanism by bridging polymer matrix was accounted for the observed stable growth of fracture inside of a carbon fiber. Carbon fiber was believed to fracture only in brittle manner. The proposed mechanism was verified by comparing single CF fracturing behavior and bulk CFRP strength at low temperatures. The high-resolution stress characterization capability of the new technique will offer a powerful tool in assisting macroscale component virtual test. It will also help optimize matrix mechanical properties towards new CFRP materials with both high stiffness and high toughness.

## Supplementary Material

Supplemental Material

## Data Availability

All data generated or analyzed during this study are included in this published article (and its Supplementary Information files).
