# Supplementary material for: Stress mapping reveals extrinsic toughening of brittle carbon fiber in polymer matrix
Source: Sci Technol Adv Mater. 2020 May 12;21(1):267–77. doi: 10.1080/14686996.2020.1752114 (PMC7269064; doi:10.1080/14686996.2020.1752114)
Supplement: Supplemental Material [file TSTA_A_1752114_SM1338.pdf]

Stress mapping reveals extrinsic toughening of brittle carbon fiber in polymer matrix

Hongxin Wang[1], Han Zhang[1]*, Kenta Goto[1], Ikumu Watanabe[1], Hideaki Kitazawa[1], Masamichi Kawai[2], Hiroaki Mamiya[1], Daisuke Fujita[1]*

1 National Institute for Materials Science, Sengen 1-2-1, Tsukuba, Ibaraki, 3050047, Japan. 2 Systems and Information Engineering, University of Tsukuba, Tsukuba, Ibaraki 305-8577, Japan.

**E-mail: ZHANG.han@nims.go.jp; FUJITA.daisuke@nims.go.jp*

Supplementary Information

I. Local modulus map for a completely broken CF

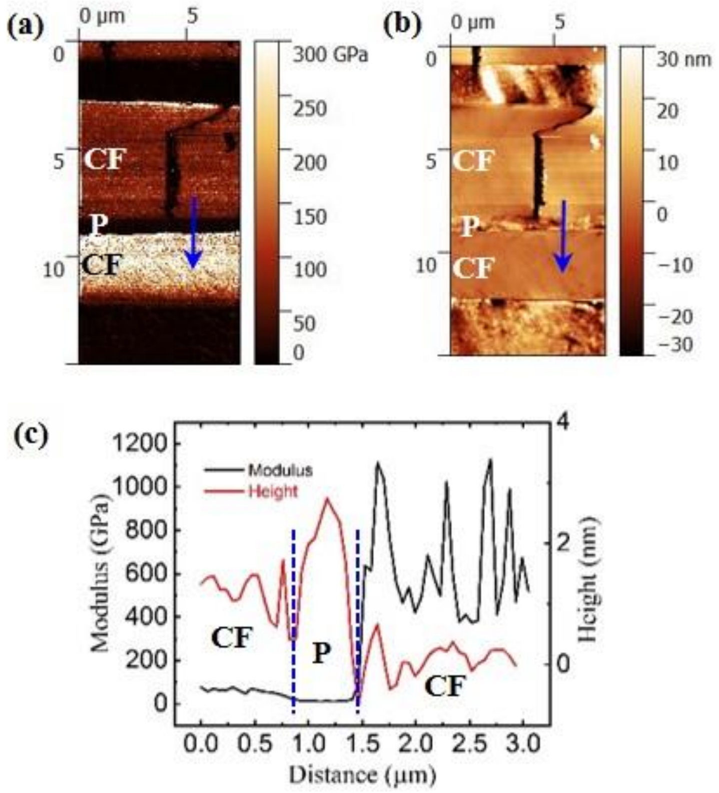

Figure S1. (a) Indentation modulus map created for the fractured CFRP with applying tensile loads. (b) Height map obtained for the same fractured CFRP simultaneously; (c) Local modulus (black) and height (red) line profiles recorded along the blue arrows marked in (a) and (b).

Local modulus of CFRP with applying tensile loads was characterized by AFM-based pinpoint indentation technique. Local modulus is affected by the intrinsic materials, induced stress and morphology of materials. As proven in the main text, local modulus map shows residual stress distribution on the CFRP surface. Thus, the detected modulus by our technique should present residual stress distribution induced by applying tensile

loads.

In figure S1(a), local modulus of the upper CF that is completely broken is lower than the lower intact CF. This phenomenon proves that stress has been released on the upper CF due to complete breakage. Figure S1(c) shows line profiles of local modulus and height along the blue arrows marked in (a) and (b) in the same region. It shows that local modulus values of both CFs are different although they are with the same height. It also confirms the fact that the observed modulus difference in the two CFs are not caused by height difference.

II. FEM Simulation of stress distribution in a polymer containing the surface-cracked CF

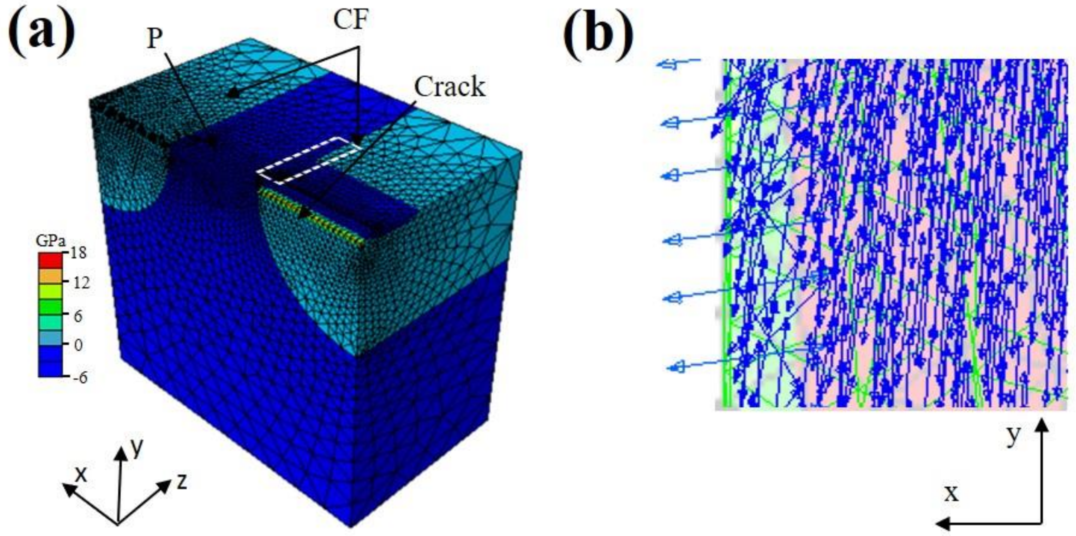

Figure S2. (a) Finite element model created to simulate the polymer matrix containing two CFs with one intact and one cracked only on its surface. (b) Direction map created for the maximal principal stress in P adjacent to CF/P interface region as marked by the white rectangle.

The elastic deformation of CFRP was simulated using a commercial finite element method (FEM) software ABAQUS 6.14 [1]. A model containing CFRP with the surface cracked CF was shown in figure S2 (a). The model is 10 x 10 x 5 mm in size and consists of 3 parts representing 2 quarter CFs and a polymer matrix. The CFs of 3.5 μm in radius are placed at a distance of 3 μm from each other in parallel to the z-axis, and one of them has a V-notch of 0.25 μm in width and depth. The interfaces of the fibers and matrix share nodes. The model is meshed with the 10-node tetrahedral element (C3D10) and the total numbers of the nodes and elements are 72261 and 49947. The carbon fiber is treated as anisotropic with material parameters as follows: $c11 = c12 = 20.99$ GPa; $c12 = 7.98$ GPa; $c13 = c23 = 9.77$ GPa; $c33 = 225.17$ GPa; $c66 = 6.51$ GPa; $c44 = c55 = 17.99$ GPa[2]. Those of the matrix are treated as isotropic with elastic modulus as 3.01 GPa and Poisson's ratio as 0.35[3]. Symmetrical boundary conditions are applied on two surfaces perpendicular to the x-axis and on one side perpendicular to the z-axis. The other side is displaced 0.0652 μm in the tensile direction. The left edge

is fixed in the y direction.

It is a fact that as soon as a fiber bridging crack is formed like the one in figure S2(a), an additional load carried by the polymer matrix is transferred to the intact CF which bridges the crack [4]. As a result, the Poisson contraction of both CF and P set up a tensile stress at their interfaces to make a stress concentration pattern as the one shown in figure 5(c) of main text. Figure 2S(b) presents the directions of the maximal principal stress on the whole polymer region. The direction along the y-axis is the same with the direction of applied tensile load. The arrows pointing left at the left edge of the model also proved the existence of tensile stress caused by Poisson contraction. Therefore, the simulation experiment proves that the strip pattern formed at the CF-P interface is due to tensile stress induced by Poisson contraction of both CF and P.

III. Simulation of a polymer matrix containing one completely broken CF by finite element model

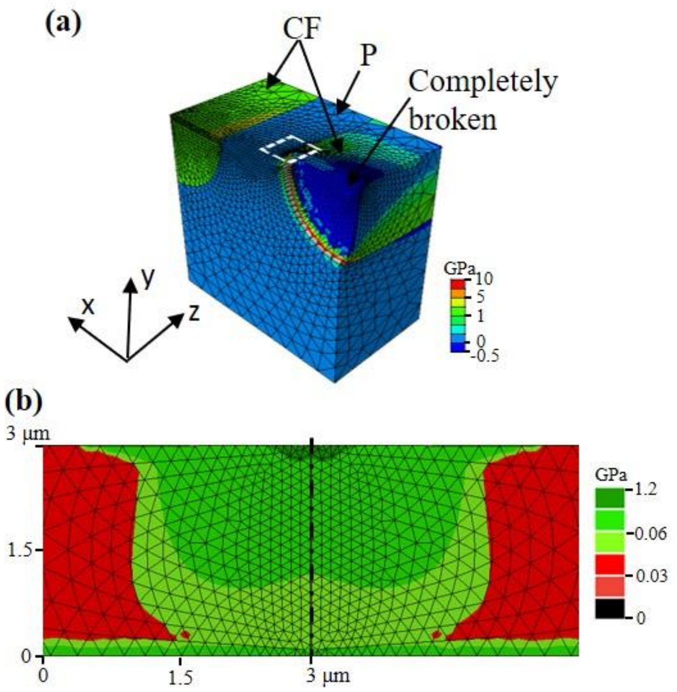

Figure S3. (a) Finite element model created to simulate a polymer matrix containing two carbon fibers with one intact and one completely broken. (b) A mirrored maximal principal stress map simulated for the polymer matrix region.

The elastic deformation of CFRP was also simulated by FEM. This model contained CFRP with the completely broken CF was illustrated in figure S3 (a). The difference with figure 2S(a) is that one CF is completely broken. The completely broken CF is also 0.25μm in width but 3.5μm in depth. Figure S3 (b) shows that maximal principal stress map simulated the polymer region. Because the upper CF is completely broken, more deformation near the fracture is caused by applying tensile loads. Stress value of polymer obtained near the fracture is higher than other part. Due to Poisson contraction

of both P and CF without fracture, the lower interface shows high stress concentration. As we know, the fact is that residual stress will be released if CF is completely fractured. Therefore, Poisson contraction of the complete broken CF cannot play a role on the upper interface. It is also confirmed by simulation in figure S3 (b). It shows that the strip pattern at the upper interface would completely disappear when the CF was completely broken. This simulation result of the completely fractured CF is different from the surface cracked CF. That case shows stress concentration on both of the interfaces.

IV. Comparison of the crack width of the fracture carbon fiber at room and lower temperatures

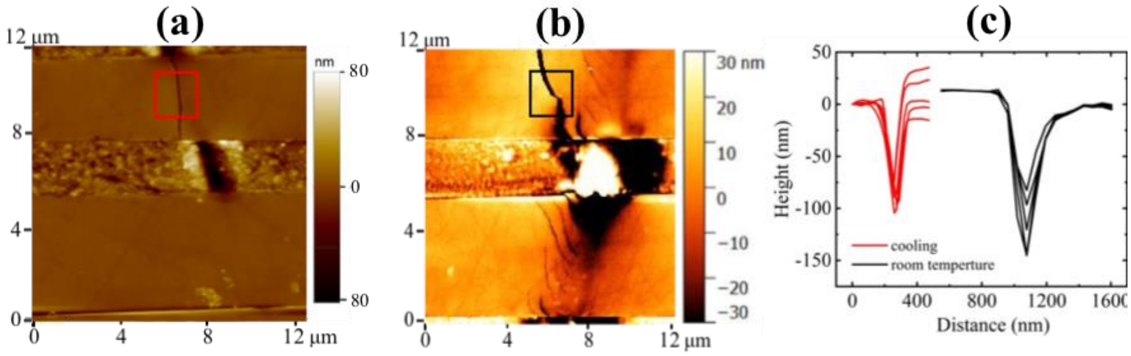

Figure S4. AFM topography of CFRP fractured (a) at low temperature (-200 °C) and (b) at room temperature (25 °C). (c) height profiles of carbon fiber from the colored rectangle in (a) and (b) respectively.

We compared the crack widths of the fractured carbon fiber at room and low temperatures. Figures S4 (a) and (b) are CFRP surface topographies from low and room temperatures, respectively. The red and black two sets of height profiles in figure S4 (c) are taken from the colored rectangular areas in figures S4 (a) and (b), respectively. We calculated the average of the five profiles and found that the crack width at low temperature is only one third of the room temperature. From the corresponding stress images in the article, it can be seen that the fractured carbon fiber under low temperature environment has lost the stress-carrying capability, while the fractured carbon fiber at room temperature still has the stress-carrying capability. That is to say, The CF was completely fractured at much lower tensile stress level at -200 ℃. Stress carrying capability of CF is completely lost even though the crack width is only 1/3 of surface fracture at room temperature.

V. Comparing Raman spectroscopy with 3-point bending test in measurement of stress

The AFM method does not provide absolute values for in-plane tensile stress. It only provides relative change in stress as governed by equation (2), using measured change in young’s modulus. We need a method of calibration to convert the relative change in stress into absolute stress value. So we used Raman spectroscopy to provide such

calibration. Raman spectroscopy measures peak shift which corresponds to stress and this conversion was done by conversion coefficient cited from literature [30]. The rationale of using this conversion coefficient from reference [30] is as follows: 1. They used the same type of carbon fiber: being both 7mm in diameter, PAN-derived and with modulus of ~350MPa; 2. They used in-situ tensile tester on a single carbon fiber to obtain this coefficient.

Alternatively, we used in-situ 3-point bending tester on CFRP specimen to justify our measurement result. The actuator force F and mid-span displacement D are recorded simultaneously while the selected carbon fiber is under Raman spectroscopy investigation. The carbon fiber selection follows the procedures of: 1. Increasing F till some carbon fibers near the outmost region of the CFRP specimen bar start to form fractures; 2. The intact carbon fiber adjacent to a fractured carbon fiber is then selected and the region directly below the fracture is investigated by Raman and later by AFM. This selection ensures that we could observe the same region before, during and after fracture taking place when actuator force F increases. This strategy relies on a prediction that fracture will propagate into the intact one from the already fractured neighbor. Figure S5 displayed two optical microscope images before and after a fracture propagate into an intact CF from a neighboring fractured CF. The white dashed lines in

the figure marked the outmost edge of the CFRP bar. The white rectangles marked the region observed by AFM and Raman during the fracture propagation. White arrow in Figure S5(b) points at the newly formed fracture below a CF with existing fracture.

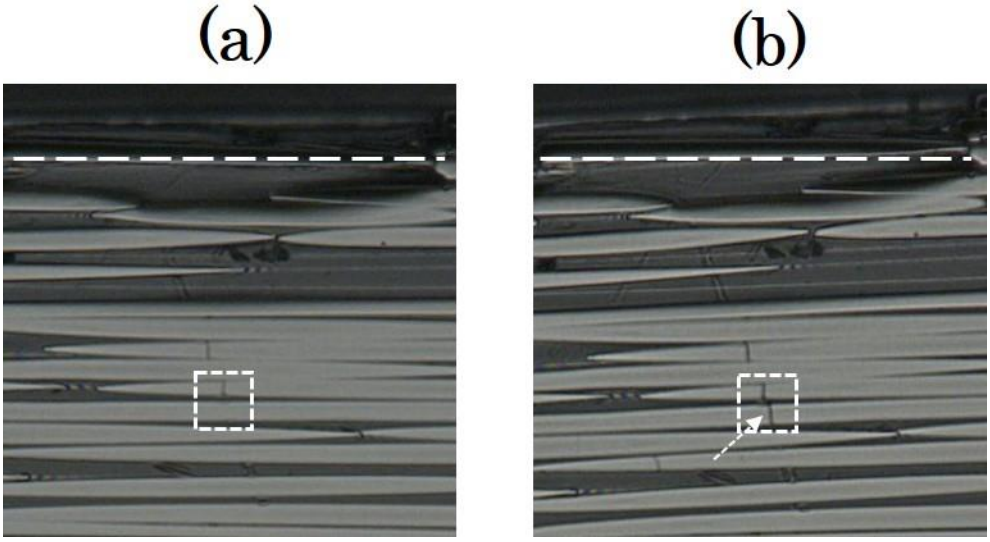

Figure S5. (a) Optical microscope image of the region near the outmost edge of the CFRP bar before fracture propagation; (b) same region after fracture propagates into the intact CF below a CF with an existing fracture. White arrow points at the newly formed fracture.

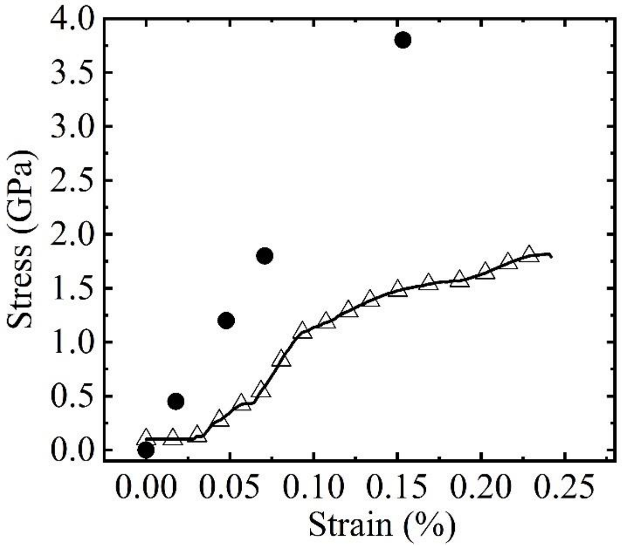

Figure S6. Stress versus strain plot during 3-point bending test of a CFRP bar. Black spheres represent local stress on a single CF surface measured by Raman microscopy. Triangles represent average stress calculated for CF bar midpoint near the outmost area.

In figure S6, *F* and *D* were converted into stress and strain for the region of the selected fiber following equation S1 and S2 as:

$$\sigma_f = \frac{3FL}{2bd^2} \qquad \text{(S1)}$$

$$\varepsilon_f = \frac{6Dd}{L^2} \qquad \text{(S2)}$$

Where σ_f and ε_f are the flexural stress and strain at specimen bar midpoint of the outmost region; L is bar span between the two support posts; b is the width of the bar; and d is the thickness of the bar.

The calculated stress is an average stress of the bar midpoint near the outmost region assuming uniform stress distribution. However, the investigated carbon fiber is adjacent to a fracture so stress higher than average is expected. This is because the load released from the existing fracture will be redistributed among neighboring intact fibers. As figure S6 shows, stress converted from Raman peak shift indicates that the stress applied on the intact fiber is about 2.5 times that of the average stress value calculated by 3-point bending test. It showed that the stress value converted from Raman spectrum is reasonable in our setup.

References

[1]. ABAQUS, version 6.14, Dassault Systèmes Simulia. *Corp. Providence,* RI, USA, (2014).

[2]. Datta, S. Ledbetter, H. & Kyono, T. Graphite-fiber elastic constants: determination from ultrasonic measurements on composite materials, in: D.O. Thompson, et al. (Eds.), Review of Progress in Quantitative Nondestructive Evaluation, Springer, New York, 1481-1488 (1989).

[3]. Luo, J. & Daniel, I. Characterization and modeling of mechanical behavior of polymer/clay nanocomposites. *Compos. Sci. Technol.* **63**, 1607-1616 (2003).

[4]. Chiang, Y. The Effects of Poisson Contraction on Matrix Cracking in Brittle-Matrix Composites. *Mech. Compos. Mater.* **37**, 207-216 (2001).
